# Hand Movement Effects on Word Learning and Retrieval in Adults

**DOI:** 10.1371/journal.pone.0053861

**Published:** 2013-01-16

**Authors:** Jessica Ciantar, Emma Finch, David A. Copland

**Affiliations:** 1 Division of Speech Pathology, The University of Queensland, Brisbane, Queensland, Australia; 2 Speech Pathology Department, Princess Alexandra Hospital, Brisbane, Queensland, Australia; 3 Centre for Functioning and Health Research, Queensland Health, Brisbane, Queensland, Australia; 4 Centre for Clinical Research, The University of Queensland, Brisbane, Queensland, Australia; UNLV, United States of America

## Abstract

The present study investigated the effect of performing an intentional non-meaningful hand movement on subsequent lexical acquisition and retrieval in healthy adults. Twenty-five right-handed healthy individuals were required to learn the names (2-syllable legal nonwords) for a series of unfamiliar objects. Participants also completed a familiar picture naming task to investigate the effects of the intentional non-meaningful movement on lexical retrieval. Results revealed that performing this hand movement immediately before linguistic tasks interfered with both new word learning and familiar picture naming when compared with no movement. These results extend previous findings of dual task interference effects in healthy individuals, suggesting that complex, non-meaningful, hand movements can also interfere with subsequent lexical acquisition and retrieval.

## Introduction

In everyday life, we frequently encounter situations that require undertaking two tasks simultaneously. Previous research using dual task paradigms has revealed that performing a secondary task may produce either positive or negative effects on the primary task, with the direction of the effect being influenced by the nature of the two tasks. In terms of research reporting detrimental dual task effects, a large body of research suggests performance on various learning and memory tasks can be negatively influenced by simultaneous performance of a secondary task [1]–[3]. This body of evidence suggests that the likelihood of the secondary task exerting negative effects on memory performance is dependent upon whether the secondary task occurs during the memory encoding or retrieval stages of the learning task, with a secondary task at the time of encoding more likely to impede memory performance [1], [3], [4]. Instructing participants to focus on the secondary task rather than the learning task produces improved performance on the secondary task while simultaneously impairing performance on the learning task, suggesting that the allocation of attentional resources between the encoding process and the secondary task is under conscious control [5].

It has been proposed that human learning and memory involves multiple cognitive systems, which act in co-operation or competition with each other [6]. One of these proposed systems related to explicit learning has been suggested to involve the frontal and medial temporal lobes, and to be dependent on attention and working memory [6], [7]. In contrast, the implicit/procedural system has been suggested to involve the striatum and to rely on automatic non-conscious processes [6], [7]. Research suggests that modulating the involvement of the explicit learning system (potentially by altering the involvement of attention and executive systems) has the capacity to alter performance on implicit learning tasks [6], [7]. Specifically, evidence suggests that minimising prefrontal cortex and/or medial temporal lobe involvement by introducing a secondary task, a distractor task, or a pharmacological agent has no effect or improves performance on tasks that rely upon the striatal learning system [6]. These findings have implications for dual task situations, as they suggest that the very nature of the tasks may determine the degree to which implicit and/or explicit learning processes are involved, potentially influencing whether the secondary task exerts a positive or negative effect on performance of the primary task. The balance between the two learning systems also has implications for language learning (especially under dual task conditions), as language learning and processing has been proposed to rely in certain conditions on implicit statistical learning mechanisms [8]–[10], with language processing closely linked to implicit sequence learning [8]–[10]. Indeed, Nemeth et al (2011) found that a sentence processing task (sentence judgement) interfered with a simultaneous probabilistic implicit sequence learning task (an alternating serial reaction time task) [10]. Thus, evidence suggests that the effectiveness of language learning can potentially be altered by introducing a dual task that modulates attention and executive processes (i.e., by manipulating the involvement of explicit learning mechanisms) or by introducing a task that involves implicit sequence learning, with the nature of the task (i.e., implicit or explicit) directly influencing the effect on the language task.

To date, the precise mechanisms behind dual task effects in general on memory encoding in healthy adults remain contentious [2]. Naveh-Benjamin and colleagues (2007) proposed that all phases of the memory encoding stage are influenced by the secondary task, with the initial encoding phase (i.e., the initial registration of information) in particular being highly susceptible to dual task interference effects [2]. Evidence suggests that the nature of the dual task may influence potential interference effects [11]. While many studies have used visual or auditory monitoring activities as the simultaneous secondary task [2], [3]–[5], several studies have also investigated the effects of more motor-based secondary tasks (e.g., button pressing, dowel balancing) as a component of the dual task paradigm [11]–[15]. In healthy right-handed individuals, performing non-meaningful movements (i.e., movements that are unrelated to the content of the speaker’s verbal output and do not facilitate the listener’s comprehension) with the right hand can interfere with simultaneous speech production [11]–[15]. It has been proposed that the interference effects may originate from shared functional neural systems controlling the execution of the linguistic and motor tasks [14], [15]. Interestingly, Lomas (1980) noted that the interference effects of linguistic activities on motor performance occurred only during specific motor tasks that involve sequenced movements without visual cues [12]. Specifically, the authors found that reciting well-known nursery rhymes produced interference effects on the simultaneous tapping of four keys in a sequence with the right fingers or arm only under conditions where participants were also unable to see the keys [12]. This observation was supported in a meta-analysis by Medland and colleagues (2002) which found that dual task interference effects varied in magnitude between studies according to the type of manual task employed, with studies that used finger tapping as the motor task differing from studies that used other motor tasks [11]. The authors proposed that the variable dual task interference effects observed during the different motor tasks may have reflected task difficulty and the allocation of attention resources, with participants more likely to allocate attention resources to the more difficult task [11].

It must be noted that a competing body of research has found that meaningful gestures may actually produce facilitatory effects in some dual task situations. Specifically, studies have revealed that performing gestures (i.e., meaningful hand movements that accompany speech) assisted participants to explain how to solve a problem while simultaneously remembering a list of unrelated items [16]–[19]. In these studies, it was found that meaningful gestures facilitated item recall. The authors proposed that this facilitatory effect was due to the gestures reducing the load on working memory [16]–[19]. There are several key elements of these studies that warrant consideration. One key feature is that the gesture was incorporated into the explanation task, rather than being a separate unrelated task. Another important element was the gesture was meaningful (rather than non-meaningful gestures that did not add to the content of the speaker’s explanation). Indeed, Cook and colleagues (2012) found that hand movements in rhythmic synchrony with speech (i.e., non-meaningful gestures) did not produce similar beneficial effects on working memory [18]. As the studies were limited to explanations and remembering lists of familiar items (e.g., letters, numbers), the effect of gesture on new word learning was not examined. There has been some evidence to suggest that meaningful gestures may actually facilitate second language learning [20], [21], however, it is unknown whether this evidence extends to nonword learning for unfamiliar concepts. Additionally, the research [20], [21] did not directly examine whether similar effects are observed with non-meaningful hand movements.

An additional stream of research into word re-learning after stroke has suggested that dual tasks may actually facilitate language learning and retrieval by acting on intentional mechanisms. Intentional mechanisms refer to the processes involved in the selection and initiation of an action from a number of competing actions [22], and are believed to be closely linked with frontal action systems of the brain (i.e., the medial frontal cortex, lateral frontal structures, and basal ganglia) [23]. Heilman and colleagues (2003) suggested that the side of the brain that the intention mechanisms are activated on depends upon the limb, lateralisation and direction of the movement [23]. In individuals with aphasia, it has been found that complex left hand movements may improve subsequent picture naming in patients with moderate to severe anomic aphasia [24], and that preactivation of the motor system by standing may facilitate lexical retrieval in patients with chronic aphasia [25]. Another study by Richards and colleagues (2002) found that patients with non-fluent aphasia demonstrated improved picture naming by performing an intentional non-meaningful complex hand action sequence prior to picture naming [26] (For clarity we have referred to the movement as complex as it involved multiple steps as opposed to simple motor movements such as repeated finger tapping). The complex hand movement involved the participant lifting the lid of a small box with his/her left hand and then pressing a button inside the box to initiate the presentation of a picture for naming [26]. It has been proposed that the facilitatory effect may have stemmed from shared functional neural resources between the linguistic and motor systems, such that preactivation of the motor system may serve to prime the linguistic system by activating shared intentional mechanisms [25], [26]. Indeed, research suggests that the pre-supplementary motor areas of the brain may control intention for both complex hand movements and word generation [24]. These findings are at odds with previous simultaneous dual task studies in healthy adults involving non-meaningful motor movements [12], [14], [15], which have found impeded performance of simple linguistic tasks paired with a simultaneous motor task, suggesting that the shared functional neural resources between the linguistic and motor systems may cause interference. Thus, further research is required to investigate the effects of simultaneous or prior complex hand movements on the complex linguistic tasks of new word learning and picture naming.

As most of the previous research into dual task effects with linguistic and non-meaningful motor movements in healthy individuals has used reciting known material (e.g., nursery rhymes), reading aloud, or simple repetition as the verbal component [15], the effects of simultaneously performing non-meaningful hand movements on new word learning are as yet unknown. New word learning necessitates the mapping of a novel word form to its referent [27]. That is the learner must link the perceptual features of the novel item to its name (phonology) and meaning (semantics) [28]. As this process is not required during reading aloud, simpler repetition or reciting known material, it is unknown whether the interference effects observed previously with simple linguistic tasks paired with non-meaningful motor movements will be observed with new word learning. Given that previous research has found that memory encoding is highly vulnerable to interference effects [2], the reliance of new word learning on the encoding of both new semantic and phonological information may cause new word learning to be particularly susceptible to dual task interference effects, especially when non-meaningful movements are included. Additionally, the established relationship between new word learning and short-term memory [29] may cause new word learning to be further vulnerable to dual task interference effects as a result of the increased cognitive requirements on short-term memory during new word learning.

In summary, evidence suggests that in healthy individuals, performing non-meaningful hand movements can interfere with simultaneous verbal tasks [11]–[15], [30], [31]. However, to date, most of the research regarding dual task effects and non-meaningful movements has targeted relatively simple verbal tasks such as reciting known material, reading aloud, or simple repetition. As a result, the effect of non-meaningful motor movements on a more complex language task such as new word learning in healthy individuals is unknown. To further complicate the issue, competing evidence suggests that pairing meaningful gestures with an explanation task improves performance on a simultaneous list-based memory task [16]–[19]. Furthermore, research in individuals with aphasia suggests that complex non-meaningful motor movements conducted immediately before linguistic tasks may actually enhance linguistic task performance by priming activation of the linguistic system [24], [25]. It is unknown whether similar facilitatory effects are observed in healthy adults when a motor movement is conducted immediately before a linguistic task. In order to address this issue, the present study examined the effects of non-meaningful complex hand movements on subsequent new word learning and word retrieval in healthy individuals. Differences between left and right hand movements were also examined. Given the non-meaningful nature of the hand movements in the present study and that previous research with healthy adults has found that simultaneous non-meaningful motor tasks can impair linguistic performance, it was hypothesized that the hand movements would interfere with lexical acquisition and retrieval. Given these previous findings in healthy individuals, it was expected that the performance of the healthy adults in the present study would differ from the results of previous aphasia research [24]–[26] involving word retrieval and complex non-meaningful hand movements, which would suggest that different possibly compensatory mechanisms are engaged in a neurologically disordered system. Given previous findings of greater interference with right hand movements in right handed individuals [12], [13], [31], it was expected that poorer new word acquisition and retrieval would be observed with right hand movements compared to left hand or no movements. To establish whether there were differential interference effects between the recall of newly learnt words compared with the recall of known words, the effects of a secondary task on subsequent naming of familiar objects was also investigated. Given the finding that memory encoding is typically more susceptible to dual task interference effects [1], [3], [4], it was hypothesized that the hand movements would result in greater declines in performance on the new word learning task compared to the familiar picture naming task.

## Methods

### Ethics Statement

The study was approved by The University of Queensland Ethics Committee and was conducted in accordance with the Declaration of Helsinki.

Participants were required to attend one testing session lasting approximately one hour. Participants completed a working memory task, a word learning task, and a familiar picture naming task. A practice task was included for both the word learning and familiar picture naming experiments.

### Participants

Twenty-five right-handed adults (12 females) aged between 18 and 32 years (mean age 22.24 years) with English as their first language participated in the study. Exclusion criteria included neurological damage or disease, degenerative disease, mental illness, learning disability, or chronic alcohol or drug abuse. Participants were recruited from the University of Queensland student body and community. Written informed consent was obtained from all participants prior to testing.

### Word Learning Task

#### Stimuli

The unfamiliar pictures used for this task consisted of 35 Finnish farm tools accessed from a previous study and used with permission of the author [28], [32], [33]. The 35 pictures were divided into seven pictures under each of the three experimental conditions and 14 practice items. The nonword names were obtained from the Australian Research Council (ARC) nonword naming database [34]. The nonword names were all two-syllable words, five or six letters in length, and adhered to the rules of written English.

#### Procedure

The learning task was administered using E-Prime software for computers (version 1.1) [35]. During the learning phase, participants were presented with a series of seven unfamiliar objects on the computer screen. Each unfamiliar object was accompanied with an auditory presentation of a nonword name via headphones. Participants were required to repeat the name of the object aloud immediately after the auditory presentation. The presentation of each object was initiated by the participant opening a wooden box with a lid and pressing a button on the mouse located within the box. This complex non-symbolic movement resembles the intentional movement utilised by Richards et al. (2002) in the aphasia rehabilitation study discussed previously [26]. Three conditions were used for the study: a right hand condition, where the participant used their right hand to open the wooden box located on their right side; a left hand condition, where the participant used their left hand to open the wooden box located on their left side; and a baseline condition, where participants were not required to make any hand movements and the task was initiated by the researcher. The order in which each condition was presented was randomised for each participant. The items used in each of the three experimental conditions were counterbalanced. A recall phase followed the learning phase, where participants were asked to recall the names of the objects by saying the learnt name aloud when presented with the objects on the computer screen. Participants were instructed to respond with ‘don’t know’ if they were unable to recall the object’s name. The learning and recall phases were repeated for a total of five trials using the same stimulus items presented in random order. All participants completed all three conditions during the session. All responses for this task were audio recorded and later transcribed for accuracy.

### Familiar Picture Naming

#### Stimuli

The familiar objects for naming were obtained from Snodgrass and Vanderwart (1980) and the International Picture-Naming Project (Szekely et al., 2004) [36], [37]. The 90 objects were of varying frequency, number of syllables, and object visual complexity as obtained from the International Picture-Naming Project [37]. The objects were split into three matched lists of 30 items for each of the conditions. There were no significant differences between the three lists in terms of frequency, number of syllables, or object visual complexity (all *p*>0.10).

#### Procedure

The familiar picture naming task was also administered using E-Prime software for computers (version 1.1) [35]. This task utilised the same three conditions (right hand, left hand, and baseline) as in the learning task. Participants used the action of opening the box and pressing the mouse button with the required hand to initiate the presentation of a series of pictures. During the baseline condition, the researcher initiated the presentation of the pictures. Participants were presented with 30 pictures of real objects for each of the three conditions. Participants were required to name the objects as quickly as possible following presentation. Participants’ responses were measured by a serial response box (model number 200A) (Psychology Software Tools) in conjunction with E-Prime software to record the latency of response time. Latency was recorded from presentation of the picture up to 2000 milliseconds. The assignment of lists to hand conditions was counter-balanced across participants.

### Working Memory

All working memory tasks were presented verbally and included (a) a wordspan forward task, requiring the participant to repeat a series of word of increasing length; (b) a digit span forward task, requiring the participant to repeat a series of numbers of increasing length; and (c) a digit span backward task, requiring the participant to repeat a series of numbers of increasing length in reverse. The number of items recalled in the correct order was recorded for each participant for all of the working memory tasks.

## Results

### Word Learning Task

**Figure 1:**
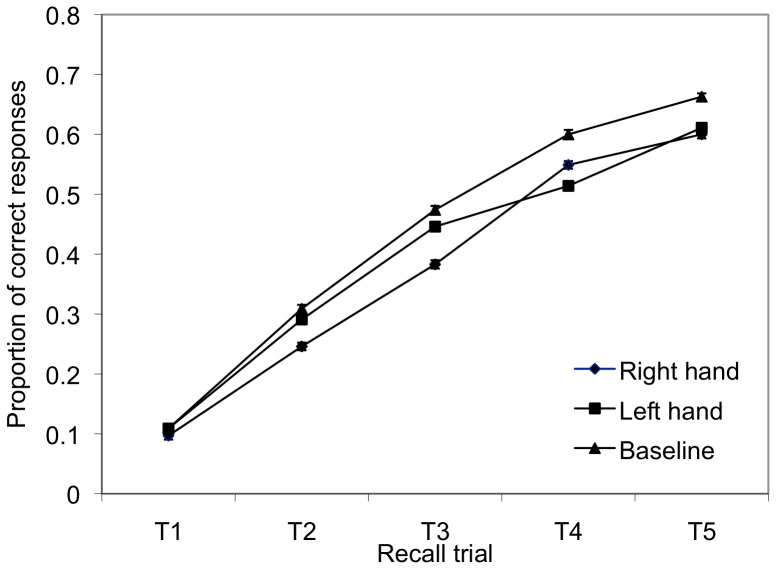
Proportion of nonword names correctly produced during the recall task. *Note.* Error bars represent within subject error bars for each condition.

Accuracy data from the learning task were entered into a mixed linear model analysis with *subject* as a random factor, *condition* (baseline, right, left) and *recall trial* (1–5) as fixed factors, and *order* (the order of the three conditions presented to each participant) and *trial within block* (the order of the items within each condition) as covariates. A bonferroni adjustment was used for calculating estimated marginal means and confidence intervals. The intra-class correlation for the model was.103. There was a significant main effect for recall trial (*F*(4, 2600) = 126.730, *p* = .000), with recall of the nonword names improving over the five learning trials, across all conditions (See Fig. 1) (Trial 1: 95% CI = .035 −.174; Trial 2 95% CI = .212 −.351; Trial 3 95% CI = .365 −.504; Trial 4 95% CI = .485 −.624; Trial 5 95% CI = .555 −.694). There was also a significant main effect for condition (*F*(2, 2600) = 3.868, *p* = .021). Overall, the baseline condition revealed the highest mean accuracy (43.1%; 95% CI = 36.5–49.7%), followed by the left hand condition (39.4%; 95% CI = 32.8–46.0%) and right hand condition (37.5%; 95% CI = 30.9–44.1%). Pairwise comparisons (Bonferroni adjusted) between the conditions were carried out, revealing a significant mean difference between the baseline and right hand condition (*p = *.019). No significant difference was found between the baseline and left hand condition (*p* = .221), or between the right hand and left hand condition (*p* = 1.00). There was no significant interaction found between condition and recall trial (*p* = .833).

### Correlations

The relationship between performance on the working memory tasks and learning task accuracy was examined using Spearman’s correlation coefficients. Accuracy data for the fifth trial (indicating learning success) was used for this analysis. As participants were required to recall the new word names following each of the five trials, analyses were conducted using the fifth recall trial (i.e., the trial that represented maximal learning) as per [46], [47], [48]. Word span and learning success were significantly positively correlated under all three conditions (Right hand: *r_s_ = *.437, *p* = .029; Baseline: *r_s_ = *.405, *p* = .045; Left hand: *r_s_* = .621, *p* = .001). Digit span forward was significantly positively correlated with learning success for the right hand (*r_s_ = *.443, *p* = .027) and baseline (*r_s_ = *.502, *p* = .011) conditions, but not for the left hand condition (*r_s_ = *.375, *p* = .065). There was no significant correlation between digit span backward and any of the three conditions (right: *r_s_ = *.269, *p* = .193; left: *r_s_ = *.231, *p* = .267; baseline: *r_s_ = *.370, *p* = .069). When overall accuracy was compared with performance on the working memory tasks, the same pattern of results emerged. There was a significant correlation between word span and overall accuracy (*r_s_ = *.554, *p* = .004), and between digit span forward and overall accuracy (*r_s_ = *.532, *p* = .006), but not between digit span backward and overall accuracy (*p*>.05).

### Familiar Picture Naming

**Figure 2:**
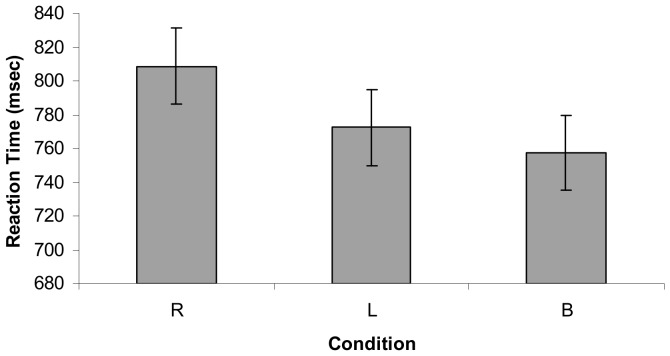
Mean reaction times in milliseconds during the familiar picture naming task. *Note.* Error bars represent the standard error for each condition.

Reaction times from the familiar picture naming task were entered into a mixed linear model analysis with *subject* as a random factor, *condition* (baseline, right, left) as a fixed factor, and *order* and *item* as covariates (see Fig. 2). A bonferroni adjustment was used for calculating estimated marginal means and confidence intervals. The intra-class correlation for the model was.139. Reaction times (RTs) greater than 1500 ms were treated as outliers and removed, resulting in 6.23% of items being excluded. The results revealed a significant main effect for condition (*F*(2, 2047) = 3.292, *p* = 0.037). Participants performed best (i.e., fastest RT) under the baseline condition (mean RT 839.80 ms; 95% CI = 764.86–914.75 ms), followed by the left hand condition (mean RT = 843.06 ms; 95% CI = 768.12–918.03 ms), and then the right hand condition (mean RT = 889.07 ms; 95% CI = 814.12–964.01 ms). Pairwise comparisons between conditions revealed no significant difference between right and left hand conditions (*p* = .097), right hand and baseline conditions (*p* = .066), or between left hand and baseline conditions (*p* = 1.00).

## Discussion

The present study examined the effect of complex non-meaningful hand movements on subsequent lexical acquisition and retrieval in healthy individuals. The findings suggest that for right-handed, healthy individuals, performing a complex non-meaningful movement immediately before linguistic tasks interferes with both lexical acquisition and retrieval of both newly learnt and previously known material. The present study used a similar experimental design (and complex motor sequence) to the aphasia rehabilitation study by Richards et al (2002) [26]. However, contrary to the facilitatory effect of the motor movement observed on later naming by Richards et al (2002) [26], the present study found that the complex motor sequence interfered with subsequent linguistic tasks. Previous reports have suggested that new word learning in healthy individuals can be used as a model for re-learning in aphasia [38]–[42]. However, if this was indeed the case, it would be predicted that the results for healthy individuals in the present study would reflect similar results to the aphasia study by Richards et al (2002) [26]. It appears that we cannot simply assume that factors influencing word retrieval in aphasia operate in a similar way to those mechanisms influencing lexical acquisition and retrieval in healthy individuals, which makes sense given the considerable differences between healthy and neurologically impaired systems. The present findings allow for the possibility that the effect seen in individuals with aphasia may arise due to facilitation of motoric aspects of speech production (post retrieval of the phonological form), however, this facilitatory effect may not arise when deficits are not present. Thus, while the present study and Richard et al (2002) employed the same complex non-meaningful hand sequence, it is possible that this task facilitated impaired post-lexical mechanisms in individuals with aphasia but interfered with lexical acquisition and retrieval mechanisms in healthy individuals.

In contrast with aphasia research, the interference effects observed in the present study more closely resembled the interference effects observed during dual task experiments with non-meaningful motor movements in young healthy adults [1]–[3]. The present study extended the results of the previous dual task research in healthy adults by revealing that interference effects can occur with complex linguistic tasks and when the non-meaningful motor task is performed immediately before the linguistic task (rather than performing the two tasks concurrently). A proposed theory to explain the dual task interference effect in the present study is that of the dual task decrement [11]. Dual task decrement stems from the idea that when carrying out a dual task, such as a motor and verbal task, both tasks need to be held in working memory with attention shared between the tasks [15]. Interestingly, the dual task decrement has been observed to be more pronounced when a right-handed task is paired with a verbal task. This phenomenon may be explained by the interference that may occur as a result of the dual processing role of the left hemisphere for both verbal tasks and right-sided movement [11]. It has been suggested that the interference between a right-sided movement and a verbal task may reflect an overlap in the neural mechanisms responsible for their processing [13], [14]. The present study lends support for this proposal, as both new word learning and familiar picture naming (which may rely upon similar neural networks, especially the inferior parietal cortex, as per Cornelissen et al., 2004 [28]) were susceptible to dual task interference effects. The present study also supports previous research demonstrating that modulating the involvement of cognitive processes associated with the explicit or procedural learning systems by introducing a secondary task can influence primary task performance [6], [7], [10].

The idea of shared neural networks between new word learning and familiar picture naming may also provide an explanation for why the two tasks experience similar interference effects despite different memory requirements. Specifically, during the picture naming task in the present study, participants were required to retrieve the name of the item under dual task conditions; no encoding of new phonological or semantic information was required. In contrast, during the new word learning task, participants were required to encode new phonological, semantic and perceptual information about the items under dual task conditions and then recall the newly learnt information. As previous research has revealed that dual task conditions at the time of encoding are more likely to impede memory performance than during other stages of the memory process [1], [3], [4], it was expected that new word learning would be more susceptible to interference effects than familiar picture naming. However, contrary to this proposal, the present study found interference effects during both new word learning and familiar picture naming.

The results of the present study differed from research that has incorporated meaningful gestures in dual task paradigms, by finding that non-meaningful gestures interfered with subsequent new word learning [16]–[19]. The present study, however, differed from this previous research on several key points, which may account for the discordant results. One important element was the gesture in the previous studies was meaningful (rather than non-meaningful as in the present study) [16]–[19], suggesting that meaningful gesture may enhance language performance while non-meaningful gesture may hinder language performance. It must also be noted that the nature of the tasks differed between the studies. The previous studies involved explaining problems with or without hand movements while simultaneously remembering lists of familiar items (e.g., letters, numbers) [16]–[19]. In contrast, the present study focused on the effects of hand movements on new word learning and familiar picture naming. Thus, one key feature of the previous studies was that the gesture was incorporated into an explanation task, rather than being a separate unrelated task. The different nature of the experimental tasks (i.e., explanation vs. new word learning) may have contributed to the differing patterns of results. Future research could examine differences between meaningful and non-meaningful gestures on new word learning in more detail.

Much of the available research regarding hemispheric asymmetries during dual tasks has utilised relatively simple verbal and motor tasks, which is in contrast with the current study [4], [6], [13], [15], [31]. Previous research has largely focussed on simpler tasks such as reciting known material, reading aloud or simple repetition as the verbal component in dual task investigations [15], [31]. These studies have provided evidence that performing a simple verbal task with concurrent right hand movements can disrupt performance. The present study extends this previous research by demonstrating that hand-related interference occurs for more complex linguistic tasks performed immediately before verbal tasks. The word learning and picture naming tasks used in the current study are also assumed to place higher demands on short-term memory than previous studies which involved simpler linguistic tasks. Given the established relationship between new word learning and short-term memory [29] it could be suggested that new word learning may be more vulnerable to dual task interference effects than simpler linguistic tasks as a result of the increased cognitive requirements on short-term memory during new word learning.

As well as the increased complexity of the linguistic tasks in the current study, the motor movements studied here can also be considered more complex than those typically used in previous studies. The motor movements required in previous research have included sequential finger tapping (tapping each finger on the hand as quickly as possible), simple finger tapping (tapping one finger on the spot), arm tapping (tapping a closed fist on a given spot), button pressing, and dowel balancing [4], [6], [13], [15], [31]. In contrast, the task required for the current study was a complex, non-meaningful movement of opening a box and pressing a button located within it [26]. Medland et al. (2002) observed that the type of motor task performed influences the size of the interference effect [11]. It was proposed that participants allocate more attention to the task that they find most difficult [11].

The current study also supports the previously established link between verbal short-term memory and the capacity to learn new words [29], [43], [44] by finding a relationship between performance on memory span forward tasks and word learning success. It has been suggested that verbal short-term memory and lexical acquisition share a common cognitive and neural network [29]. Working memory also plays an important role in dual task effects. When carrying out a dual task, such as the motor and linguistic tasks in this study, both tasks need to be held in working memory with attention shared between the tasks [15]. Therefore, it can be assumed that better performance on memory tasks may be predictive of performance on dual tasks given the increased working memory capacity. The results of the current study support this proposal, as demonstrated by a positive correlation between the working memory tasks (word and digit span forward) and performance on the dual tasks utilised in the study (new word learning and familiar picture naming combined with the complex non-symbolic movement).

The lack of correlation between digit span backward and word learning in the current study is also of interest. This finding may be explained by the difference in the functions required for memory span forward and backward tasks [45]. The word and digit span forward tasks used in this study involve the maintenance of information only, whereas the digit span backward task requires both the maintenance and active manipulation of information. It may be inferred that working memory tasks involving the simple maintenance of information are more closely related to the processes involved in new word learning studied here.

It must be noted, however, in the present study that while right hand movements but not left hand movements differed significantly from baseline during the recall of newly learnt words, there was no significant difference between left and right hand movements on the task. The absence of a significant interference effect with the left hand during the new word learning task may have been an artefact of statistical power. It is possible that with an increased sample size, a statistically significant interference effects might be observed between these conditions or alternatively interference effects may have been observed for both hand movements compared to baseline (no movement).

### Conclusion

In conclusion, the present study demonstrated that performing complex, non-meaningful, hand movements interfered with subsequent lexical acquisition and retrieval in healthy individuals. This finding is consistent with previous dual task studies involving simultaneous tasks and non-meaningful movements in healthy individuals. However, the results of the current study appear to be contradictory with findings of intention treatment and facilitation effect found in individuals with aphasia (despite using a similar experimental design). This discrepancy suggests that the facilitation effect found in aphasia may be more due to motor speech facilitation (i.e., the facilitation of body parts involved in speech production) than lexical retrieval or alternatively that normal mechanisms were not being tapped into in this treatment. The present study also conflicts with the results of dual task studies in healthy adults involving meaningful movements, suggesting that the meaningfulness (or lack of meaningfulness) of the motor movements may be an important factor. The present study supports the established link between new word learning and short-term memory and the premise that performance on short-term memory tasks may be predictive of new word learning capacity. Future research is required to fully understand the interactions between intentional non-meaningful movements and language performance.
